# Supplementary material for: Thoracic Inlet in Cervical Spine CT of Blunt Trauma Patients: Prevalence of Pathologies and Importance of CT Interpretation
Source: Tomography. 2022 Nov 19;8(6):2772–83. doi: 10.3390/tomography8060231 (PMC9680416; doi:10.3390/tomography8060231)
Supplement: Supplementary file 1 [file tomography-08-00231-s001.zip › tomography-2008139-supplementary.pdf]

Table S1: CT findings of thoracic inlet pathologies and their corresponding chest radiographic findings

Pathologies	CT findings	Corresponding chest radiographic findings
**Traumatic**		
**Mediastinal fat stranding**	Fat haziness within mediastinum, usually surrounding vascular structures	Abnormal mediastinum
**Mediastinal hemorrhage**	Discrete slightly hyperattenuating collection within mediastinum, potentially surrounding vascular structures	Abnormal mediastinum
**Pneumomediastinum**	Extraluminal air within soft tissues of mediastinum	Same as CT
**Pulmonary contusion**	Fluffy, ill-defined airspace opacification in the lungs not respecting anatomical boundaries; may spare subpleural region	Patchy pulmonary opacification
**Pulmonary laceration**	Pulmonary cavity filled with blood, air or both and surrounded by pulmonary contusion	Usually not seen
**Pneumothorax**	Extraluminal air collection inside pleural cavity, often in curvilinear shape	Same as CT
**Pleural fluid**	Fluid of variable attenuation within pleural cavity, usually in dependent portion	Apical cap
**Extrapleural hematoma**	High-attenuating collection, often biconvex shape and at site of rib fracture, displacing extrapleural fat inwardly to the lung	Apical cap
**Rib fracture**	Cortical break – without or with displacement – of ribs	Same as CT
**Clavicle fracture**	Cortical break – without or with displacement – of clavicle	Same as CT

**Acromioclavicular dislocation**	Loss of normal articulation between acromion and distal clavicle	Same as CT
**Scapular fracture**	Cortical break – without or with displacement – of scapula	Same as CT
**Significant nontraumatic**		
**Mediastinal vascular dilation**	Dilation of artery ($\geq$1.5 times of expected normal caliber) – often a branch of aortic arch – within superior mediastinum	Abnormal mediastinum
**Pulmonary nodule(s)**	Rounded or oval-shaped discrete noncalcific solid abnormality inside the lung parenchyma	Same as CT
**Pulmonary micronodules**	Tiny pulmonary nodules of less than 4 mm; having various shapes and arrangements including<br>- Tree-in-bud (V- or Y-shaped micronodules in lung periphery)<br>- Centrilobular (micronodules in centrilobular distribution; not touching pleurae/fissures)<br>- Other appearances	Usually not seen
**Groundglass opacity**	Increased lung opacity without obscuration of lung markings	Usually not seen
**Groundglass nodule(s)**	Similar to pulmonary nodule(s) but attenuation is lower (not obscure lung markings)	Usually not seen
**Cavity**	Air-filled collection with soft-tissue rims within lung parenchyma	Same as CT
**Atelectasis**	Airspace parenchymal opacification with loss of volume	Same as CT

**Septal thickening**	Thickened interlobular septa of lung parenchyma	Usually not seen
**Active tuberculosis**	Cavity, tree-in-bud and centrilobular pulmonary micronodules, pulmonary nodule(s), groundglass opacity in uni- or bilateral lung apices	Same as CT
**Pulmonary malignancy**	Irregular-shaped pulmonary nodule(s) or mass(es) with or without spiculated border	Same as CT
**Foreign body**	Abnormal structure of various attenuation and shape usually within esophagus or trachea	Same as CT
**Malignant bone lesions**	Lucent or sclerotic lesions associated with soft tissue masses	Same as CT
**Non-significant**		
**Parenchymal scars**	Linear or curvilinear pulmonary opacities associated with distortion of pulmonary architecture	Usually not seen
**Calcifications**	High attenuation foci or nodule(s)	Same as CT
**Bronchiectasis**	Dilation of bronchi greater than their accompanying pulmonary artery branches	Usually not seen
**Emphysema**	Increased lucency of pulmonary parenchyma without definable walls; without or with stretched lung markings	Same as CT
**Blebs/bulla**	Focal lucency within pulmonary parenchyma with thin walls	Usually not seen
**Benign bone lesions**	Bone islands; lucent lesions with complete and smooth sclerotic rims	Same as CT

Table S2: Performance of portable trauma chest radiography in identification of thoracic inlet abnormalities* using cervical spine CT as a reference standard

	True positive	False positive	False negative	True negative	Sensitivity (95% CI)	Specificity (95% CI)
**Overall performance**	**90**	**7**	**159**	**129**	**36.14 (30.17-42.45)**	**94.85 (89.68-97.91)**
**All traumatic findings**	**52**	**51**	**40**	**242**	**56.52 (45.78-67.83)**	**82.59 (77.76-86.76)**
**Abnormal mediastinum**	0	4	11	370	0.00 (0-28.49)	98.93 (97.28-99.71)
**Pulmonary contusion**	17	24	23	321	42.50 (27.04-59.11)	93.04 (89.83-95.49)
**Pneumothorax**	9	0	40	336	18.37 (8.76-32.02)	100 (98.91-100)
**Extrapleural hematoma**	1	48	1	335	50.00 (1.26-98.74)	87.47 (83.73-90.61)
**Rib fracture**	12	10	12	351	50.00 (29.12-70.88)	97.23 (94.96-98.66)
**Clavicle fracture**	7	16	1	361	87.50 (47.35-99.68)	95.76 (93.20-97.56)
**Scapular fracture**	0	3	1	381	0 (0-97.50)	99.22 (97.73-99.84)
**All significant nontraumatic findings**	**8**	**8**	**82**	**287**	**8.89 (3.92-16.77)**	**97.29 (94.73-98.82)**
**Pulmonary nodule(s)**	4	5	27	349	12.90 (3.63-29.83)	98.59 (96.73-99.54)
**Active tuberculosis**	3	5	10	367	23.08 (5.04-53.81)	98.66 (96.89-99.56)

**Destructive bone lesions**	1	0	0	384	100 (2.50-100.00)	100 (99.04-100.00)
**Nonsignificant findings**	13	12	211	149	5.80 (3.13-9.72)	92.55 (87.34-96.09)
**Parenchymal scars**	20	3	54	308	27.03 (17.35-38.61)	99.04 (97.21-99.80)
**Calcifications**	4	0	11	370	26.67 (7.79-55.10)	100 (99.01-100)
**Bronchiectasis**	0	1	24	360	0 (0.00-14.25)	99.72 (98.47-99.99)
**Emphysema/blebs**	1	0	92	292	1.08 (0.03-5.85)	100 (98.74-100)

*Listed entities include those deemed potentially visible on chest radiography and were present in our study cohort

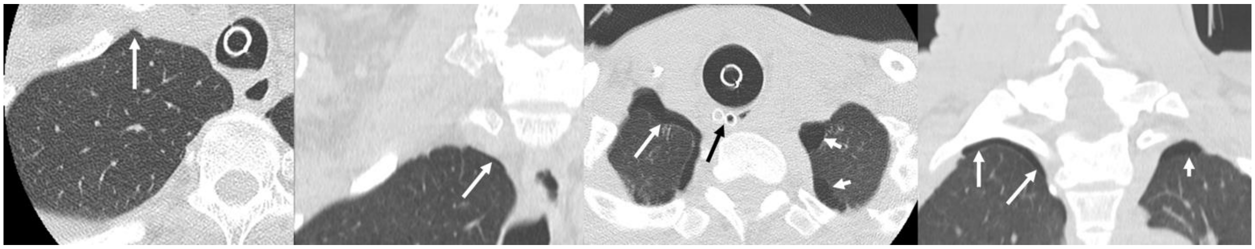

Figure S1: Pneumothorax and subpleural bleb/bulla. Axial and coronal-reformatted CT images in lung window of two different patients show minimal right pneumothorax (long arrows). A few small subpleural blebs/bullae (short arrows) in the left lung are convex toward the lung parenchyma. Perception of minimal pneumothorax may be improved with coronal reformation. Black arrow = esophagogastric tube coiled within the thoracic esophagus.

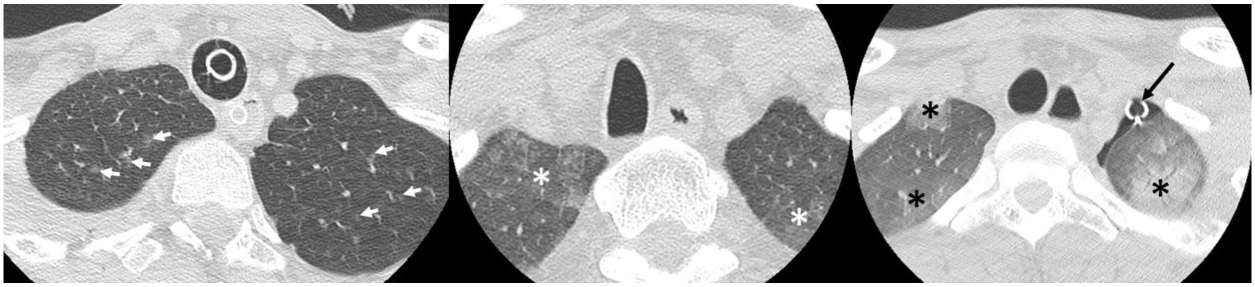

Figure S2: Pulmonary contusion. Axial CT images in lung window of three different patients show different features and extent of pulmonary contusion, which include small ill-defined centrilobular groundglass opacities (short arrows), ill-defined groundglass opacities superimposed with interlobular septal thickening (crazy paving pattern; white stars), and "fluffly" groundglass opacities with consolidations (black stars). Black arrow = intercostal drainage tube with left pneumothorax.

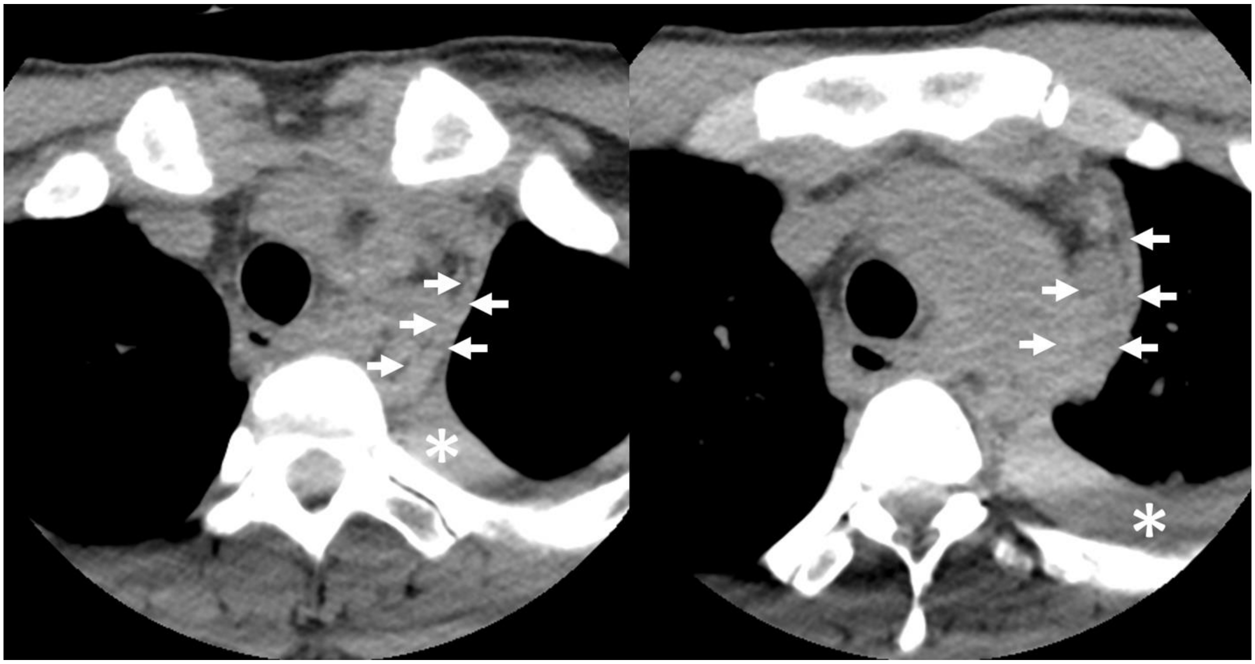

Figure S3: Mediastinal hemorrhage. Axial CT images in soft-tissue window show ill-defined isoattenuating bands or streaks (arrows) interspersed within superior mediastinal fat, abutting aortic arch and its branches. There is a small amount of isoattenuating left pleural fluid (stars), representing hemothorax.

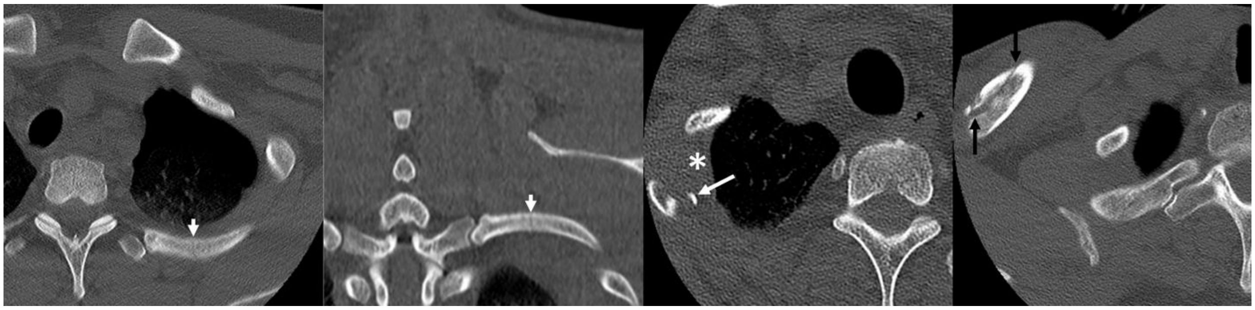

Figure S4: Rib and clavicle fractures. Axial and coronal-reformatted CT images in bone window/algorithm of three different patients show a nondisplaced fracture of the left posterior rib (short white arrows), a displaced rib fracture fragment (long white arrow) associated with an extrapleural hematoma (star), and a nondisplaced comminuted right clavicle fracture (black arrows). These can be easily overlooked on a cervical spine CT due to their subtlety and location at the edge of image series.

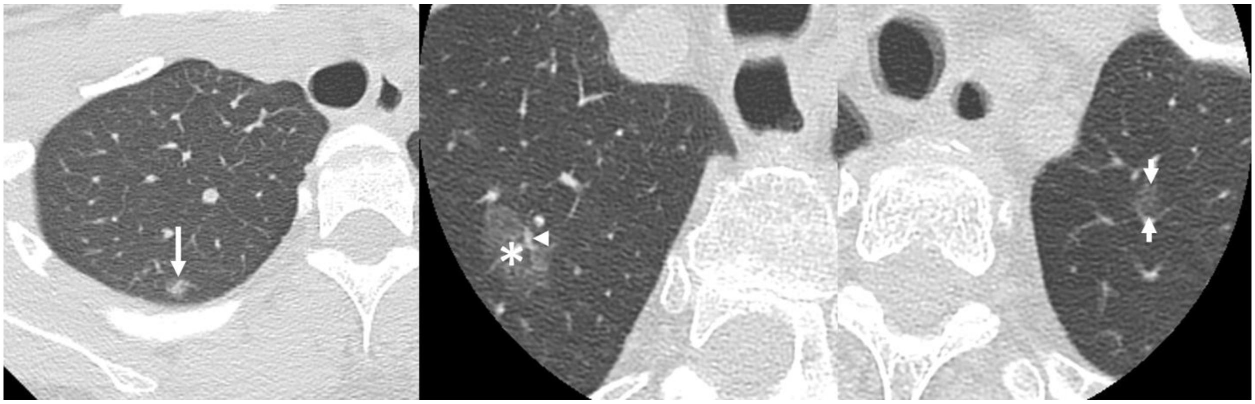

Figure S5: Subsolid pulmonary nodules. Axial CT images in lung window of three different patients reveal three subsolid pulmonary nodules. Two are part-solid groundglass nodules (long arrow and star), in which an arrowhead indicates a solid portion of the larger nodule. Short arrows point at a pure groundglass nodule.

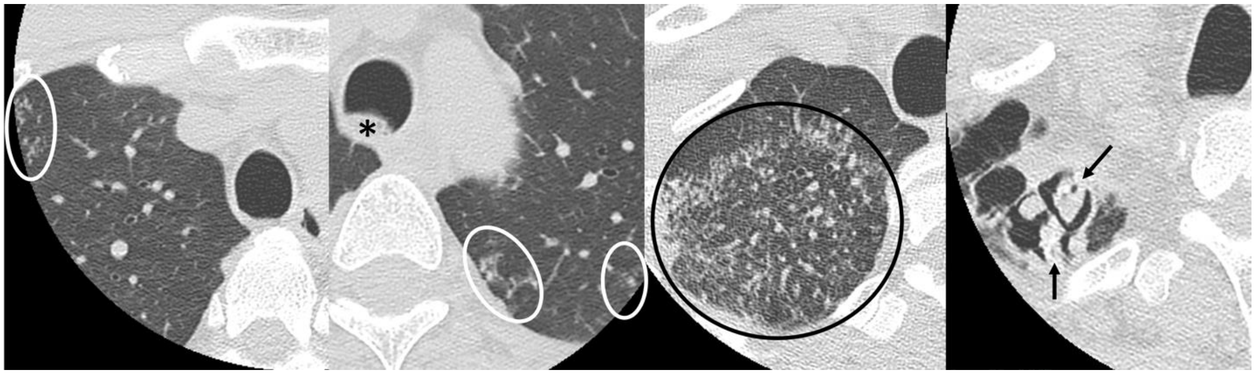

Figure S6: Pulmonary micronodules and cavities. Axial CT images in lung window of four different patients demonstrate small clusters of tree-in-bud opacification and centrilobular nodules (white oval), a larger area of the same abnormality (black oval), and a few air-filled cavities containing rounded contents (arrows). The black star represents fluid in the distal trachea.
